# Correlation analysis of coagulation factor level and chronic low-grade inflammation in patients with type 2 diabetes mellitus complicated with ischemic stroke

**DOI:** 10.1515/med-2025-1275

**Published:** 2026-03-20

**Authors:** Li Weng, Yan Wang, Dejuan Li

**Affiliations:** Department of Endocrinology, People’s Hospital of Qijiang District, Chongqing, China; Department of Neurology, People’s Hospital of Qijiang District, Chongqing, China

**Keywords:** T2DM, IS, coagulation factors, plasma inflammatory factors, Pearson correlation coefficient

## Abstract

**Objectives:**

To analyze differences in coagulation function and plasma inflammatory factors between type 2 diabetes mellitus (T2DM) patients with and without ischemic stroke (IS), and explore related mechanisms.

**Methods:**

50 patients with T2DM complicated with IS (experimental group, EG) and 50 patients with simple T2DM (control group, CG) were retrospectively included, while 30 healthy volunteers served as the blank group (BG). The expression levels of plasma interleukin-6 (IL-6), tumor necrosis factor-α (TNF-α), and nuclear factor-κB (NF-κB) in the EG were significantly elevated as against the CG and the BG (p<0.05).

**Results:**

As against the CG and the BG, activated partial thromboplastin time (APTT) and prothrombin time (PT) levels were significantly reduced, while the plasma fibrinogen (FIB) and D-Dimer (DD) levels were significantly elevated in the EG; The incidence of postoperative complications in the EG (13.46 %) was significantly reduced as against the CG (21.15 %) (p<0.05). IL-6 was notably negatively correlated with APTT (p*=*0.002, *r=*−0.432); it was extremely significantly negatively correlated with PT (p*=*0.000, *r=*−0.536); it was notably positively correlated with plasma FIB content (p*=*0.001, *r=*0.445).

**Conclusions:**

The coagulation function and peripheral blood inflammatory markers in patients with T2DM complicated by IS were significantly different compared to those in patients with T2DM alone and healthy volunteers. The correlation between inflammatory markers and coagulation indicators (*e.g.,* the negative correlation between IL-6 and APTT/PT) identified in this study holds important clinical significance. It provides new insights for thrombosis risk stratification and personalized antithrombotic treatment in T2DM patients. Monitoring the levels of inflammatory markers may optimize thrombosis prevention strategies and enhance the precision of clinical diagnosis and treatment.

## Aim

It explored the correlation between inflammatory factors and coagulation function in T2DM patients, especially T2DM patients with IS. The study aims to understand the role and impact of inflammatory response and coagulation function in T2DM and its complications by comparing the differences and correlations between peripheral blood inflammatory factors and coagulation function indicators in different groups (T2DM patients with IS, simple T2DM patients, and healthy volunteers).

## Core tip

The study retrospectively included 50 patients with T2DM combined with IS (experimental group, EG) and 50 patients with T2DM alone (control group, CG). In addition, 30 healthy volunteers who came to the hospital for physical examination during the same period were included as the blank group (BG). It compared the inflammatory factors and coagulation function indicators of peripheral blood in three groups of patients, and analyzed the relationship between different inflammatory factors and coagulation function using Pearson correlation coefficient. It suggested that inflammatory response may be accompanied by enhanced blood coagulation function, increasing the risk of thrombosis.

## Introduction

In patients with type 2 diabetes mellitus (T2DM), ischemic stroke (IS) is a kind of common complication, which seriously affects the quality of life (QoL) and health status of patients [1]. Past studies have shown that diabetes mellitus (DM) patients not only have typical microvascular complications such as vascular endothelial dysfunction and vascular inflammation, but also have an increased risk of thrombosis and coagulation abnormalities [2], [3], [4]. Moreover, the association between T2DM and stroke is also widely recognized, but the specific mechanism is not fully understood. Persons with T2DM and IS often show a variety of symptoms, including but not limited to neurological dysfunction, movement disorder, language disorder, and cognitive dysfunction [5], 6]. These symptoms not only have a serious impact on the patients’ daily life, but also bring a heavy burden to the family and society. The difficulty of treatment also increases, because these patients often have a variety of potential health problems, such as cardiovascular disease, hypertension, hyperlipidemia, which need to be comprehensively considered and personalized treatment plan [7]. When formulating the treatment plan, the medical team should not only consider the patients’ main diseases, but also comprehensively evaluate and manage these concomitant diseases, so as to reduce the risk of complications and improve the treatment effect. This requires the collaboration of multidisciplinary teams, including experts in cardiology, endocrinology, nutrition, and other departments, to jointly develop and implement the best treatment strategy [8]. In addition, patients’ lifestyle interventions, such as diet adjustment, exercise planning, and psychological support, also play a key role in comprehensive treatment. The implementation of tailored and all-encompassing care plans has been demonstrated to obviously elevate the overall QoL for patients, addressing their unique needs and preferences [9].

In terms of related mechanism research, the pathogenesis of T2DM with IS involves many aspects. First, DM patients have chronic hyperglycemia, which can lead to vascular endothelial function damage, increased platelet activation, and increased vascular permeability, thereby increasing the risk of thrombosis and cerebrovascular disease [10], [11], [12]. Chau et al. [13] described the frequency and time characteristics of acute cerebral infarction in persons with T2DM, and discussed the influence of blood glucose fluctuation on the prognosis of acute cerebral infarction. It was concluded that the greater the blood glucose fluctuation of patients, the more risk factors for stroke, and the worse the short-term prognosis. Secondly, DM patients are often accompanied by microvascular complications, such as microvascular lesions and neuropathy, which may further aggravate the damage of cerebral vessels and the occurrence of IS [14]. In a study by Huang et al. [15], 120 individuals diagnosed with T2DM were selected based on specified inclusion and exclusion criteria to investigate the contributing factors to acute IS among this population. The results suggested that high-sensitivity C-reactive protein (hs-CRP) and lipoprotein-associated phospholipase A2 (Lp-PLA2) were independent contributing factors of acute IS in persons with T2DM and potential markers of stroke risk. In addition, coagulation factors play a major role in thrombosis and inflammatory response, and the chronic low-grade inflammation may be one of the key factors that lead to the increased risk of vascular injury and stroke [16], 17]. It is of great significance to explore the association between coagulation factor levels and body micro inflammation in persons with T2DM and IS for in-depth understanding of the pathogenesis of this complication and improving the level of diagnosis and treatment.

Therefore, in-depth study of the pathophysiological mechanism of T2DM with IS is essential to develop effective prevention and treatment strategies. The aim of this article is to analyze the level of coagulation factors in persons with T2DM and IS, and to explore the correlation between them and the chronic low-grade inflammation. Through the comprehensive evaluation of coagulation factors and micro inflammatory indicators, it is expected to reveal the pathophysiological mechanism of T2DM with IS, offering novel foundations and concepts to enhance clinical diagnostics and therapeutic approaches.

## Materials and methods

### Subjects

Fifty subjects with T2DM and IS (experimental group, EG) and 50 subjects with simple T2DM (control group, CG) admitted to the Department of Endocrinology/Neurology of People’s Hospital of Qijiang District from December 2022 to January 2024 were retrospectively included. In addition, 30 healthy volunteers who came to People’s Hospital of Qijiang District for physical examination at the same time were included as the BG. The sample size determination considered previous similar studies (with ≥30 cases per group to meet the test power) and performed a power analysis using G*Power 3.1. Taking the correlation between interleukin-6 (IL-6) and activated partial thromboplastin time (APTT) (effect size *r*=0.4) as an example, the minimum sample size was calculated to be 38 cases. The sample size in each group of this study exceeded this threshold, ensuring a test power of ≥0.85. Statistical tests on the baseline data (age, gender, body mass index (BMI)) of the three groups showed no significant differences (p>0.05), indicating that although the blank group (BG) had a smaller sample size than the EG/CG, the comparability between groups was good, and no significant bias was introduced into the results.

A total of 68 candidate patients with T2DM complicated by IS were screened during the study period, with 18 excluded for the following reasons: i. coexisting other autoimmune diseases (n=7); ii. previous stroke with significant sequelae (n=5); iii. missing clinical data that could not be analyzed (n=4); iv. coexisting mental illness (n=2). Ultimately, 50 cases were included in the EG. Simultaneously, 72 candidates with T2DM alone were screened, with 22 excluded (including six pregnant/lactating women, 8 cases with incomplete clinical data, 6 cases with severe infections, and two for other reasons). A total of 50 cases were included in the CG. The inclusion criteria for the BG were healthy individuals matched for age and gender with the EG/CG, with no history of diabetes, cardiovascular disease, or infectious diseases. A total of 30 cases were included in the BG.

Inclusion criteria: the duration of DM was within 6 years; the glycated hemoglobin content is less than 10 %; it meets the diagnostic criteria for DM patients formulated by the WHO (Table 1); the patients were all adults; patients able to communicate with medical staff normally; it meets the diagnostic criteria for DM complicated with IS formulated by the WHO (Table 2).

**Table 1:** Diagnostic criteria of T2DM.

Symptom	Typical symptoms	Random blood glucose	Fasting blood glucose	Oral glucose tolerance test (OGTT)	Diagnostic criteria
Typical symptoms of DM, such as polydipsia, polyuria, polyphagia, and weight loss excluding other reasons	Presence	≥11.1 mmol/L (200 mg/dL)	≥7.8 mmol/L (140 mg/dL)	Not applicable	DM can be diagnosed according to either random blood glucose or fasting blood glucose
Typical symptoms of DM, but blood glucose does not meet the diagnostic criteria	Presence	<11.1 mmol/L (200 mg/dL)	<7.8 mmol/L (140 mg/dL)	≥11.1 mmol/L (200 mg/dL) 2-h blood glucose	OGTT results should meet the standard
There was no clinical manifestation of typical DM, but the OGTT results were abnormal	Absence	/	/	DM can be diagnosed when both 1-h and 2-h blood glucose are ≥11.1 mmol/L (200 mg/dL), or 2-h blood glucose is ≥11.1 mmol/L (200 mg/dL) when OGTT is repeated, or fasting blood glucose is ≥7.8 mmol/L (140 mg/dL)	DM can be diagnosed if patients meet the criteria of OGTT results
There was no clinical manifestation of typical DM, and the OGTT results were normal	Absence	/	/	Both 1-h and 2-h blood glucose were <11.1 mmol/L (200 mg/dL), and fasting blood glucose was <7.8 mmol/L (140 mg/dL)	Patients do not meet any diagnostic criteria and DM can’t be diagnosed

**Table 2:** Diagnostic criteria of T2DM with IS.

Diagnostic indicators	Diagnostic criteria
History of DM	1. DM has been diagnosed; 2. Random blood glucose ≥ 11.1 mmol/L (200 mg/dL); 3. Fasting blood glucose ≥ 7.0 mmol/L (126 mg/dL)
History of stroke	1. A history of acute onset of nervous system ischemic damage; 2. CT or MRI suggested ischemic brain damage
Clinical symptoms	1. Sudden neurological deficit (such as paralysis, speech disorder, sensory disorder) lasts for more than 24 h; 2. There are symptoms of IS
Neuroimaging findings	1. Cerebral ischemic lesions in head CT or MRI; 2. Angiography revealed carotid or cerebral artery stenosis or obstruction
Pathophysiological findings	1. Blood examination suggested that glucose concentration in arterial blood increased; 2. The measured value of glycosylated hemoglobin increased; 3. Neuropathological changes are associated with DM

Exclusion criteria: patients with other autoimmune diseases; patients with obvious sequelae from previous stroke; clinical data were missing and could not be analyzed normally; patients with mental illness; pregnant or lactating women.

### Data collection

(1) A specially assigned person was responsible for collecting the gender, age, height, and weight of the included subjects. BMI was calculated according to the equation.
(2) The levels of inflammatory factors in peripheral blood of subjects were collected, including IL-6, TNF-α, nuclear factor-κB (NF-κB).
(3) The coagulation function indexes of subjects were collected, including plasma fibrinogen (FIB), APTT, prothrombin time (PT), D-Dimer (DD).

### Quality control

In this article, a retrospective investigation was initiated to examine the relationship between coagulation factor levels and body micro inflammation in subjects with T2DM and IS. The study design included the description of the design type, participant recruitment methods, sample size, data collection methods, etc., and detailed the retrospective characteristics of the study, including the source and time range of data collection. In the process of sample selection, the source, inclusion criteria, and exclusion criteria of samples were clarified, and the process of sample matching was described. In terms of data collection, standard data collection tools and measurement methods were used, and the methods to deal with missing data, outliers, or incomplete data were explained. To control the influence of bias and confounding factors, the possible influencing factors were discussed, and the corresponding control measures were explained. In terms of the reliability and interpretation of the results, the reliability and accuracy of the research results were described in detail, and the possible bias in the interpretation of the results was discussed. In addition, the results of this article were compared with those of other similar studies, and the consistency and distinctions were discussed. The limitations of the study were also identified and described, and suggestions were made based on the findings, and the direction of future research was discussed. Through these quality control measures, the transparency and credibility of the design, methods, and results of the study were ensured.

### Statistical methods

Statistical analysis was performed using *SPSS 19.0*. Quantitative data were expressed as mean ± standard deviation (x ± s), and categorical data were expressed as percentages (%). For the comparison of coagulation function indicators (APTT, PT, FIB, DD) and inflammatory markers (IL-6, tumor necrosis factor-α (TNF-α), NF-κB) among the three groups, one-way analysis of variance (One-Way ANOVA) was used, as each group consisted of independent samples and each indicator was measured only once across different groups. The earlier reference to repeated measures ANOVA was incorrect and is hereby corrected. Pairwise comparisons between groups were performed using the LSD-t test, with Bonferroni correction applied to adjust the threshold for the p-value to 0.05/3≈0.017, in order to control the Type I error rate. Pearson correlation coefficient was used to analyze the correlation between coagulation function indicators and inflammatory markers, with the correlation coefficient r and corresponding p-value calculated. The strength of correlation was determined based on |*r*| values (|r|<0.3 indicates weak correlation, 0.3≤|*r*|<0.7 indicates moderate correlation, and |*r*|≥0.7 indicates strong correlation). Power analysis was conducted using G*Power, with a significance level set at *α*=0.05 and a power of 1−*β*=0.8 to ensure the reliability of the results. A difference was considered statistically significant if the corrected p-value was <0.017.

### Ethical approval statement

This study has been approved by the Medical Ethics Committee of People’s Hospital of Qijiang District, Chongqing. The research was conducted in accordance with the ethical principles outlined in the *Declaration of Helsinki*.

### Consent to participate

Informed consent was obtained from all individual participants included in the study.

## Results

### Contrast of general information of subjects

**Figure 1:**
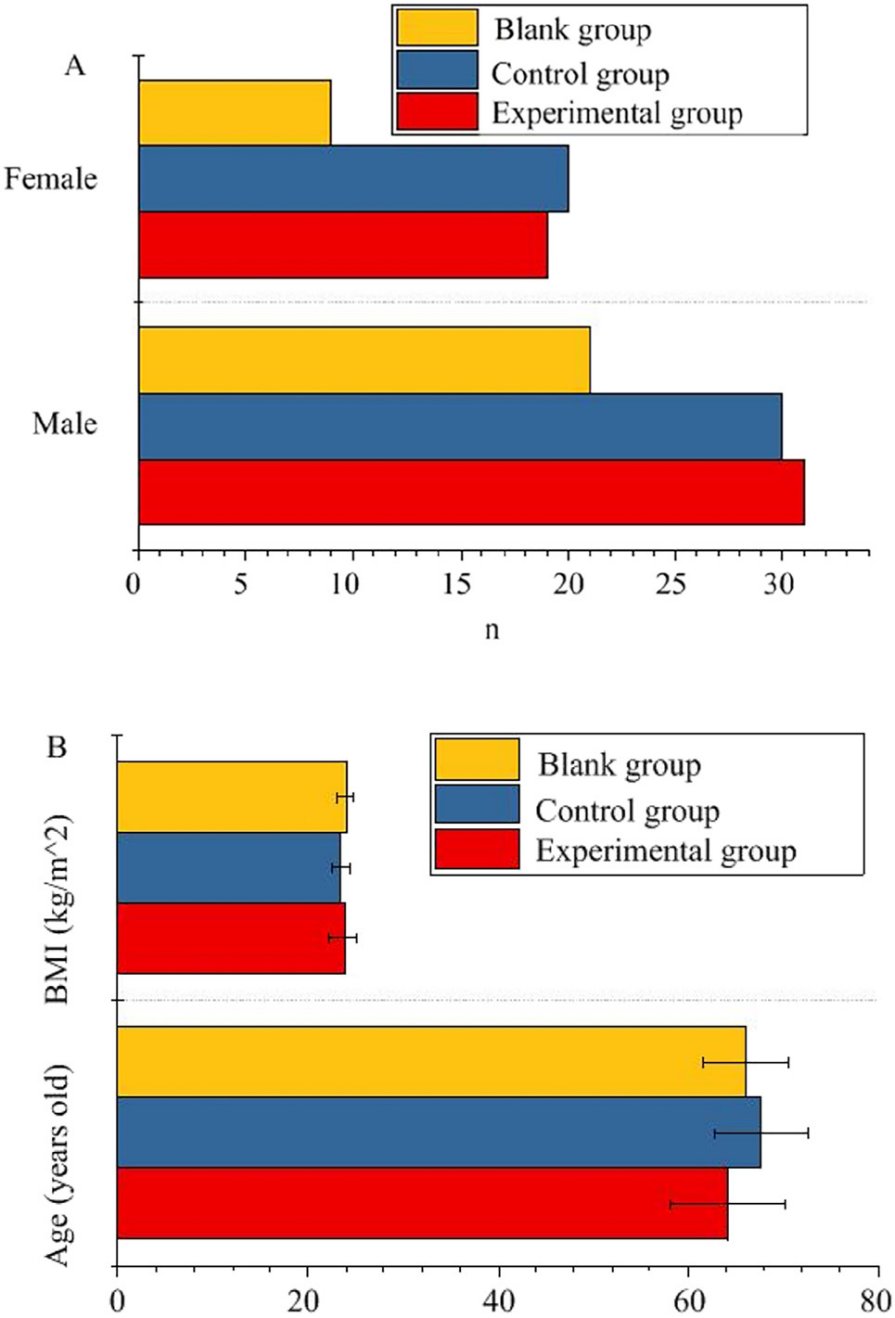
Comparison of general data of research objects. (A) Gender; (B) age and BMI.

There was no visible distinction in age, sex, and BMI of the subjects (p*>*0.05) (Figure 1).

### Contrast of coagulation function indexes among the three groups

**Figure 2:**
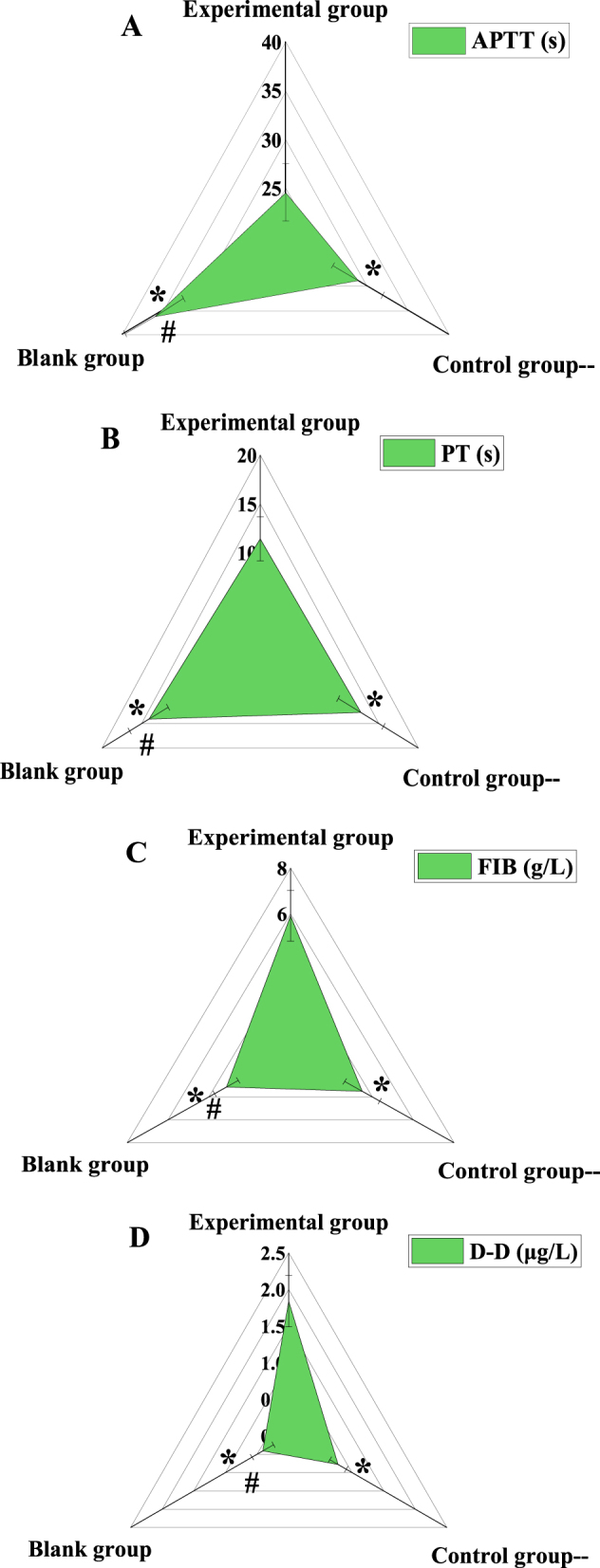
Comparison of coagulation function indexes among three groups. (A) Plasma FIB; (B) APTT; (C) PT; (D) DD. Note: * indicates as against the EG, # means as against the CG, p*<*0.05.

This study conducted a comparative analysis of coagulation function indicators among the EG, CG, and BG (Figure 2). For APTT, the EG had a value of 24.61 ± 2.95 s, the CG had 28.83 ± 3.06 s, and the BG had 36.14 ± 3.57 s. A significant decrease in APTT was observed in the EG compared to both the CG BG (p<0.05). For PT, the EG showed 11.42 ± 2.25 s, the CG had 12.73 ± 2.86 s, and the BG had 14.08 ± 2.43 s, again demonstrating that the EG was significantly lower than the other two groups (p<0.05). The plasma FIB levels were 5.91 ± 1.11 g/L in the EG, 3.51 ± 0.86 g/L in the CG, and 3.14 ± 0.58 g/L in the BG, with a significant increase observed in the EG (p<0.05). For DD, the EG had 1.84 ± 0.35 μg/L, the CG had 0.78 ± 0.13 μg/L, and the BG had 0.41 ± 0.17 μg/L, with the EG significantly higher than the other two groups (p<0.05). The CG showed a reduction in APTT and PT, and an increase in FIB and DD compared to the BG, exhibiting a similar trend (p<0.05).

### Contrast of inflammatory factor levels of subjects

**Figure 3:**
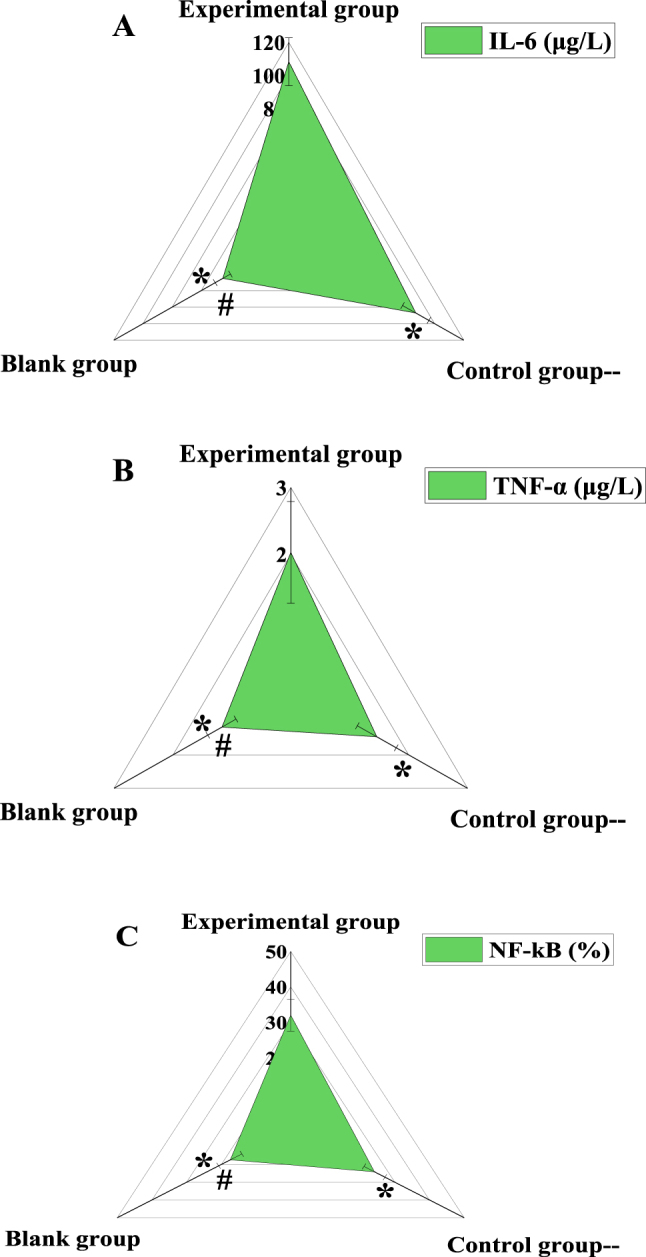
Comparison of inflammatory marker levels among the three groups. (A) Interleukin-6 (IL-6); (B) tumor necrosis factor-α (TNF-α); (C) nuclear factor-κB (NF-κB). Note: * indicates as against the EG, # means as against the CG, p*<*0.05.

Analysis of the inflammatory marker levels in the three groups revealed the following (Figure 3). For IL-6, in the EG, 31 male subjects had an IL-6 level of 108.38 ± 14.46 μg/L, and 19 female subjects had a corresponding value. In the CG, 30 male subjects had an IL-6 level of 87.11 ± 9.03 μg/L, and 20 female subjects were included. In the BG, 21 male subjects had an IL-6 level of 45.25 ± 5.21 μg/L. Overall, the plasma IL-6 level in the EG was significantly higher than in the CG and BG (p<0.05). For TNF-α, the EG had a value of 2.03 ± 0.76 μg/L, the CG had 1.46 ± 0.33 μg/L, and the BG had 1.17 ± 0.24 μg/L, with the EG showing significantly higher levels than the other two groups (p<0.05). For NF-κB, the EG had 32.06 ± 4.51 %, the CG had 24.15 ± 3.02 %, and the BG had 17.41 ± 3.35 %, again showing a significant increase in the EG (p<0.05). The CG also showed a significant increase compared to the BG (p<0.05).

### Correlation between coagulation function indicators and inflammatory factors in subjects

**Figure 4:**
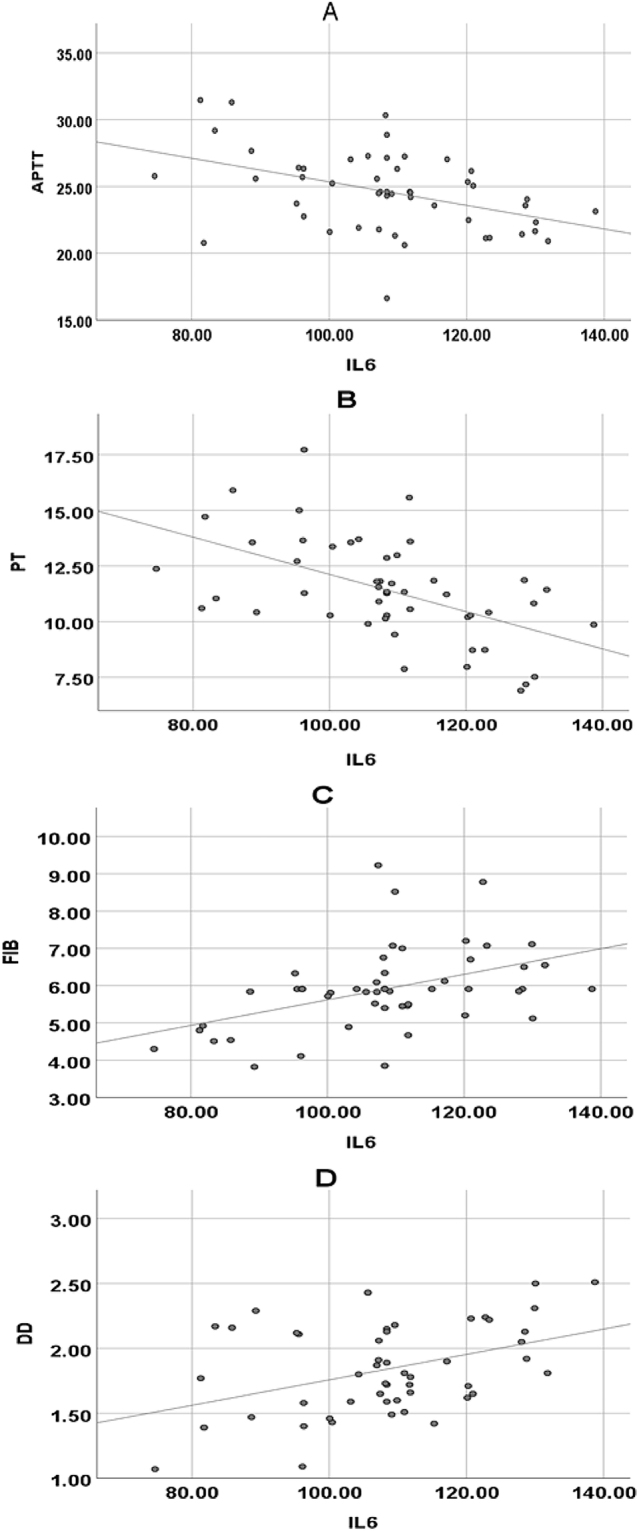
Pearson correlation analysis of IL-6 and FIB, APTT, PT, DD in subjects. (A) IL-6 and APTT; (B) IL-6 and PT; (C) IL-6 and FIB; (D) IL-6 and DD.

The Pearson correlation coefficient was used to analyze the relationship between coagulation function and inflammatory markers (Figure 4). IL-6 showed a significant negative correlation with APTT (p=0.002, *r*=−0.432), indicating a moderate negative correlation. This suggests that as IL-6 levels increase, APTT tends to shorten significantly. IL-6 was also strongly negatively correlated with PT (p=0.000, *r*=−0.536), indicating a strong negative correlation, meaning that higher IL-6 levels are associated with shorter PT. IL-6 exhibited a significant positive correlation with FIB (p=0.001, *r*=0.445), demonstrating a moderate positive correlation, indicating that an increase in IL-6 levels is associated with an increase in FIB concentration. TNF-α showed a significant negative correlation with APTT (p=0.001, *r*=−0.443), representing a moderate negative correlation. NF-κB was significantly negatively correlated with APTT (p=0.005, *r*=−0.394), indicating a moderate negative correlation, and NF-κB was significantly positively correlated with FIB (p=0.027, *r*=0.313), suggesting a weak positive correlation.

**Figure 5:**
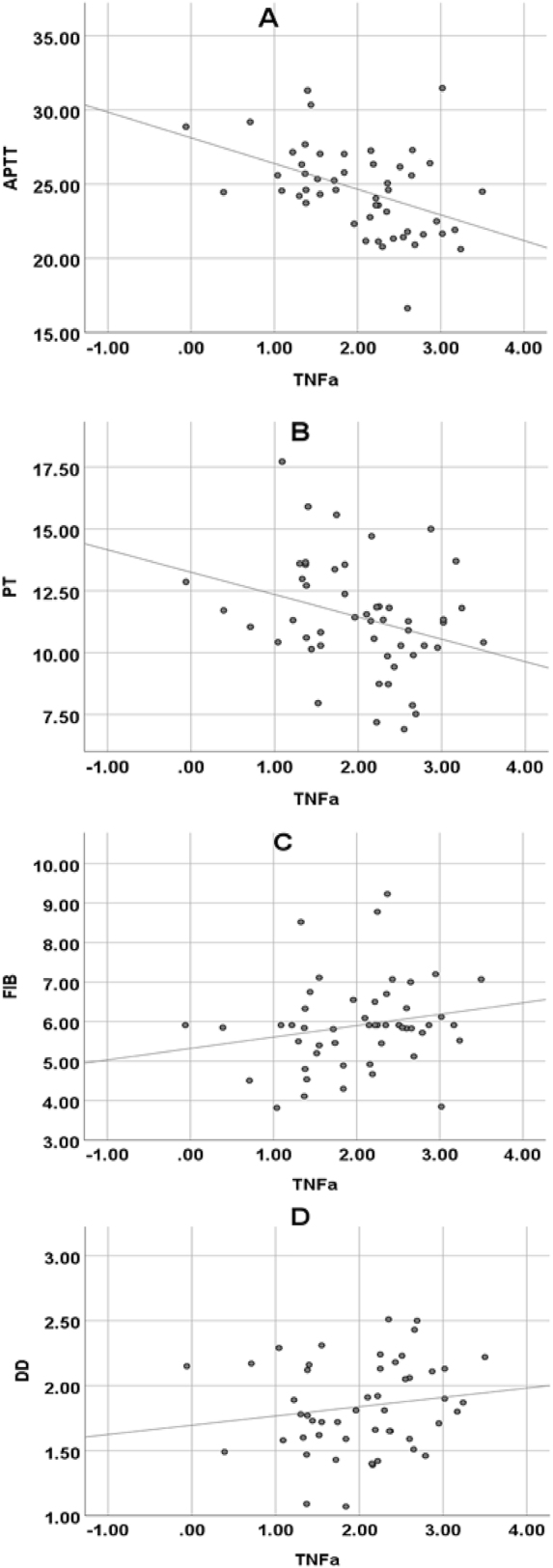
Pearson correlation analysis of TNF-α and FIB, APTT, PT, DD in subjects. (A) TNF-α and APTT; (B) TNF-α and PT; (C) TNF-α and FIB; (D) TNF-α and D-D.

Pearson correlation coefficient was used to analyze the relationship between TNF-α and plasma FIB, APTT, PT, and DD. TNF-α was markedly negatively correlated with APTT (p*=*0.001, *r=*−0.443) and PT (p*=*0.032, *r=*−0.303); it was not markedly correlated with plasma FIB content (p*=*0.174, *r=*0.195) and DD content (p*=*0.280, *r=*0.156) (Figure 5).

**Figure 6:**
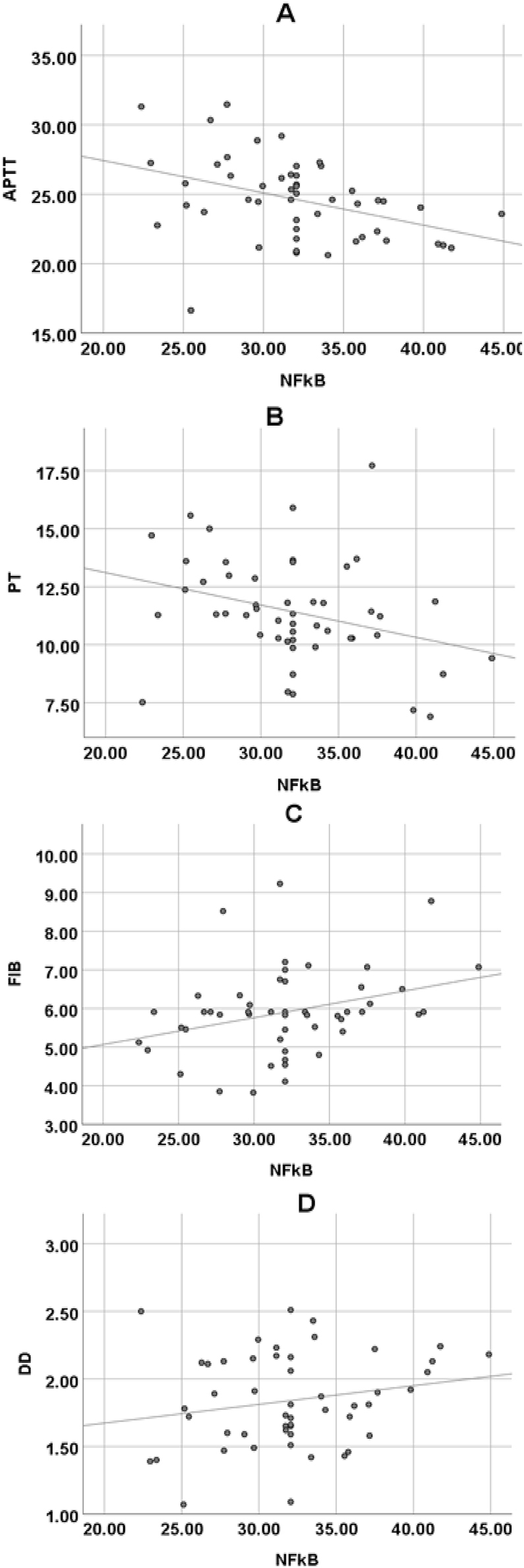
Pearson correlation analysis of NF-κB and FIB, APTT, PT, DD in subjects. (A) NF-κB and APTT; (B) NF-κB and PT; (C) NF-κB and FIB; (D) NF-κB and D-D.

Pearson correlation coefficient was used to analyze the relationship between NF-κB and plasma FIB, APTT, PT, and DD. NF-κB was markedly negatively correlated with APTT (p*=*0.005, *r=*−0.394) and PT (p*=*0.029, *r=*−0.310); it was markedly positively correlated with plasma FIB content (p*=*0.027, *r=*0.313); it was not markedly correlated with DD content (p*=*0.161, *r=*0.201) (Figure 6).

## Discussion

T2DM is a chronic disease characterized by a long-term rise in blood glucose levels, which can lead to a variety of complications, including cardiovascular disease and cerebrovascular disease. IS is brain tissue damage caused by insufficient blood supply to cerebral vessels [18]. For patients with both T2DM and IS, the goals of treatment usually include controlling blood glucose levels, managing blood pressure and blood lipid levels, and taking measures to prevent further cardiovascular and cerebrovascular events, which may include drug treatment, changing lifestyle (such as diet and exercise), and regular medical examination and monitoring [19], [20], [21]. The pathogenesis of T2DM and IS involves a variety of factors, including metabolic abnormalities, vascular lesions, inflammatory reactions, etc., so in-depth study of the pathophysiological mechanism of T2DM with IS is essential to develop effective prevention and treatment strategies [22]. This article retrospectively included 50 subjects with T2DM and IS (EG) and 50 subjects with simple T2DM (CG). In addition, another 30 healthy volunteers were included as the BG. The peripheral blood inflammatory factors IL-6, TNF-α, NF-κB and coagulation function indicators FIB, APTT, PT, DD of the subjects were compared. Pearson correlation coefficient was used to analyze the correlation between coagulation function indexes and inflammatory factors. There was no visible distinction in age, sex, and BMI of the subjects (p*>*0.05). This result shows that the basic characteristics between these groups are similar in the early stage of the study, which is beneficial for subsequent data analysis and result interpretation [23].

The decrease of APTT and PT may indicate the overactivation or abnormality of the coagulation mechanism in the patient, resulting in the blood being too easy to clot, which may increase the risk of thrombosis. The elevation of plasma FIB and DD levels is generally considered as an indicator of the activation of the coagulation system and thrombosis *in vivo* [24], 25]. APTT and PT were markedly lower, while plasma FIB and DD were markedly higher in the EG as against the CG and the BG (p*<*0.05). It revealed that the coagulation function of T2DM patients with IS was markedly different from that of patients with simple T2DM and healthy volunteers. Liu et al. [26] included 120 persons with T2DM as the research subjects, analyzed the contributing factors of IS in depth, and found that the levels of hs-CRP and Lp-PLA2 were markedly correlated with patients complicated with IS. This article found that plasma IL-6, TNF-α, and NF-κB in the EG were markedly higher as against the CG and the BG, while those were markedly higher in the CG as against the BG (p*<*0.05). This is similar to the research results of Liu j et al., which shows that peripheral plasma IL-6, TNF-α, NF-κB in T2DM patients with IS are much higher as against simple T2DM and healthy volunteers.

Pearson correlation coefficient was used to analyze the relationship between coagulation function and inflammatory markers. IL-6 showed a significant negative correlation with APTT (p=0.002, *r*=−0.432), indicating a moderate negative correlation. This suggests that as IL-6 levels increase, APTT tends to shorten more noticeably. IL-6 also exhibited a highly significant negative correlation with PT (p=0.000, *r*=−0.536), indicating a strong negative correlation, meaning that higher IL-6 levels are associated with shorter PT. IL-6 was significantly positively correlated with FIB (p=0.001, *r*=0.445), demonstrating a moderate positive correlation, meaning that an increase in IL-6 levels is accompanied by an increase in FIB concentration. TNF-α showed a significant negative correlation with APTT (p=0.001, *r*=−0.443), representing a moderate negative correlation. NF-κB was significantly negatively correlated with APTT (p=0.005, *r*=−0.394), indicating a moderate negative correlation, and NF-κB was significantly positively correlated with FIB (p=0.027, *r*=0.313), suggesting a weak positive correlation. These results further elucidate the intrinsic relationship between inflammatory markers and coagulation function [27]. To further explore the underlying biological mechanisms, IL-6 plays a crucial role in regulating coagulation function. On one hand, IL-6 can drive the upregulation of fibrinogen. Fibrinogen is a key protein in the coagulation process, and its elevated levels accelerate the coagulation cascade, leading to shortened APTT and PT, which aligns with the negative correlations observed between IL-6 and APTT, PT in our study [28]. On the other hand, IL-6 can activate endothelial cells, rendering them pro-coagulant. Under normal conditions, endothelial cells maintain a balance between anti-coagulation and pro-coagulation. However, under the influence of IL-6, endothelial cells express more pro-coagulant factors, such as tissue factor, disrupting this balance and enhancing coagulation. This is consistent with the argument put forward by Attiq et al. [29], who suggested that high-sensitivity C-reactive protein (hs-CRP) serves as a marker for evaluating systemic inflammation and predicting coronary artery disease risk based on its peak plasma levels. Both hs-CRP and IL-6 are related to inflammation and coagulation function. While hs-CRP, as a classic marker of inflammation, reflects the inflammatory state and impacts the coagulation process, IL-6 may be positioned further upstream in the inflammatory regulation cascade. Not only can IL-6 directly affect coagulation function, but it may also indirectly influence coagulation by modulating other inflammatory mediators [30]. It was hypothesized in this study that IL-6 may have a synergistic mechanism with other inflammatory markers, such as TNF-α and NF-κB. IL-6 might enhance the pro-inflammatory and pro-coagulant effects of TNF-α and NF-κB, while TNF-α and NF-κB could, in turn, promote the secretion of IL-6, creating a positive feedback loop that exacerbates both the inflammatory response and coagulation changes. Future research is needed to explore the synergistic interactions between these inflammatory markers to better understand the complex relationship between inflammation and coagulation function.

This study has the following limitations: First, it employed a retrospective design. Although statistical methods were used to verify the correlation between inflammatory markers and coagulation function, causal relationships cannot be directly inferred. For instance, the association between IL-6 and coagulation markers may be influenced by other confounding factors, such as blood glucose fluctuations or concomitant medication use. Second, the sample source was limited to a single-center dataset, which may introduce selection bias due to regional population characteristics. Additionally, the sample size for the healthy control group was relatively small (n=30). Future studies should expand the sample size and include multi-center data. To further validate the research hypothesis, the following exploratory studies are recommended: i. A prospective cohort study to dynamically monitor IL-6 levels in T2DM patients and the occurrence of thrombotic events over time, to clarify the causal relationship between inflammation and coagulation activation; ii. Mechanistic studies using cell or animal models to explore the regulatory mechanisms of the IL-6/NF-κB pathway on coagulation factor expression; iii. Multi-center clinical studies to assess the generalizability of IL-6 as a thrombosis risk biomarker. These studies will contribute to a deeper understanding of the pathological processes in T2DM complicated by IS from both causal inference and mechanistic perspectives.

## Conclusions

A total of 50 patients with T2DM complicated by IS (EG), 50 patients with T2DM alone (CG), and 30 healthy volunteers as the blank group (BG) were retrospectively included in this study. The peripheral blood inflammatory markers and coagulation function indicators of the participants were compared, and Pearson correlation coefficients were used to analyze the relationships between the indicators in order to understand the role and impact of coagulation function and inflammatory response in T2DM and its complications. The results showed significant differences in coagulation function and peripheral blood inflammatory markers between patients with T2DM complicated by IS, patients with T2DM alone, and healthy volunteers. Inflammatory markers such as IL-6, TNF-α, and NF-κB were significantly correlated with several coagulation markers, suggesting that inflammation may be associated with enhanced coagulation function, thereby increasing thrombosis risk.

Regarding clinical recommendations, given the significant correlation between IL-6 and coagulation parameters, IL-6 monitoring can be considered as a supplementary tool for thrombosis risk assessment in T2DM patients, particularly those with concomitant IS. For individuals with elevated IL-6 levels, enhanced antithrombotic interventions should be implemented. Based on the mechanism by which IL-6 drives pro-coagulation, the use of IL-6 receptor antagonists or other anti-inflammatory drugs in improving hypercoagulable states could be explored. Due to the complex interplay between blood glucose, inflammation, and coagulation, a multidisciplinary approach involving endocrinology, neurology, and hematology is recommended to optimize the comprehensive management of T2DM patients with IS.

As for future research directions, large-scale prospective studies are needed to validate the predictive value of inflammatory markers such as IL-6 for thrombotic events in T2DM patients. Further investigation is required to explore the specific mechanisms by which IL-6, TNF-α, and other inflammatory markers regulate endothelial pro-coagulant factors through pathways such as NF-κB. Basic and clinical research should be conducted to assess the effects of anti-inflammatory drugs like canakinumab on improving coagulation function and reducing the risk of stroke recurrence. Longitudinal studies are essential to determine the impact of chronic inflammation on the progression of cerebrovascular complications and the long-term effectiveness of combined anti-inflammatory and antithrombotic strategies. These studies will provide more precise clinical strategies for inflammation-related thrombosis prevention.
